# Young people’s differential vulnerability to criminogenic exposure: Bridging the gap between people- and place-oriented approaches in the study of crime causation

**DOI:** 10.1177/1477370817732477

**Published:** 2018-01-14

**Authors:** Per-Olof H Wikström, Richard P Mann, Beth Hardie

**Affiliations:** University of Cambridge, UK; University of Leeds, UK; University of Cambridge, UK

**Keywords:** Artificial neural network modelling, crime causation, person–environment interaction, Peterborough Adolescent and Young Adult Development Study (PADS+), Situational Action Theory, space–time budget

## Abstract

The overall purpose of this study is to contribute to bridging the gap between people- and place-oriented approaches in the study of crime causation. To achieve this we will explore some core hypotheses derived from Situational Action Theory about what makes young people crime prone and makes places criminogenic, and about the interaction between crime propensity and criminogenic exposure predicting crime events. We will also calculate the expected reduction in aggregate levels of crime that will occur as a result of successful interventions targeting crime propensity and criminogenic exposure. To test the hypotheses we will utilize a unique set of space–time budget, small area community survey, land-use and interviewer-led questionnaire data from the prospective longitudinal Peterborough Adolescent and Young Adult Development Study (PADS+) and an artificial neural network approach to modelling. The results show that people’s crime propensity (based on their personal morals and abilities to exercise self-control) has the bulk of predictive power, but also that including criminogenic exposure (being unsupervised with peers and engaged in unstructured activities in residential areas of poor collective efficacy or commercial centres) demonstrates a substantial increase in predictive power (in addition to crime propensity). Moreover, the results show that the probability of crime is strongest when a crime-prone person is in a criminogenic setting and, crucially, that the higher a person’s crime propensity the more vulnerable he or she is to influences of criminogenic exposure. Finally, the findings suggest that a reduction in people’s crime propensity has a much bigger impact on their crime involvement than a reduction in their exposure to criminogenic settings.

Two core criminological findings are that the distribution of crime in the population is highly skewed – a small minority of people are responsible for a majority of crimes (for example, Piquero et al., 2007: 17–19; Wikström, 1990; Wolfgang et al., 1972) – and that crime events (and particular types of crime events) tend to be concentrated in space and time – sometimes referred to as *hotspots* (for example, Sherman et al., 1989; Weisburd et al., 2012; Wikström, 1991). Criminological theories (and research) tend to focus on the role of either people (propensities) or places (environmental inducements) in crime causation; rarely do they consider how both may be explained (and analysed) within a common theoretical framework. And yet arguably neither can be adequately explained (and studied) without taking the other into consideration (Wikström and Treiber, 2016).

Discussing individual (people) and community (place) oriented research traditions in criminology, Albert Reiss Jr. convincingly argued quite some time ago that ‘more is to be gained by linking those traditions than by their continued separate development and testing’ (1986: 29). To bridge this divide and integrate key insights about the role of people and places in crime causation, and to move beyond (at times) unfruitful competition and conflict between person- and place-oriented approaches, we argue that (proper) situational analysis should form the foundation of criminological theory. However, ‘researchers have thus far done little to develop a systematic situational perspective’ (LaFree and Birkbeck, 1991: 73), a statement that is still largely valid.

To overcome the neglect of (proper) situational analysis and advance knowledge about crime and its causes, we specifically maintain that criminology needs at its core (i) an adequate action theory^1^ that helps integrate key insights from people- and place-oriented approaches (Kroneberg et al., 2010; Wikström, 2004), and (ii) data and methodologies that better allow us to study the role of and test hypotheses about the person–environment interaction (Wikström et al., 2012a). In this study we aim to help bridge the gap between people- and place-oriented approaches and advance knowledge – theoretically, methodologically and empirically – about the person–environment interaction in crime causation. Guided by Situational Action Theory, and utilizing a unique set of space–time budget, small area community survey, land-use and interviewer-led questionnaire data from the prospective longitudinal Peterborough Adolescent and Young Adult Development Study (PADS+) and an artificial neural network approach to modelling, we will explore some hypotheses about what makes people crime prone and makes places (settings) criminogenic, and specifically test the interactional hypothesis that young people vary in their vulnerability to criminogenic exposure depending on their level of crime propensity.

## Situational Action Theory

Situational Action Theory (SAT) is a general, dynamic and mechanism-based theory of crime causation (for example, Wikström, 2006, 2010, 2014). It analyses crime as acts of *rule-breaking* and stresses the importance of the *person–environment interaction* and the need to properly understand and explicate the *action mechanism* that links people and their immediate environments to their actions, such as acts of crime. SAT insists that people are the *source* of their actions (people perceive, choose and execute their actions) but that the *causes* of their actions are situational (people’s particular perception of action alternatives, process of choice and execution of action are triggered and guided by the relevant input from the person–environment interaction).

Whereas most major criminological theories (such as control and opportunity theories^2^) seem to work under the (human nature) assumption that people’s action choices are mainly driven by self-interest (Agnew, 2014), SAT asserts that humans are essentially rule-guide creatures and society (social order) is based on shared rules of conduct (Wikström, 2010). SAT defines and analyses acts of crime as *moral actions*, that is, ‘actions which are guided by value-based rules of conduct specifying what is the right or wrong thing to do (or not do) in response to particular motivations in particular circumstances’. Acts of *crime* are specifically defined as ‘breaches of rules of conduct stated in law’, and this is what all acts of crime, in all places, at all times, have in common. SAT asserts that the same process that explains why people follow or break rules of law should also explain why they follow or break other kinds of moral rules (for example, informal rules of conduct).

**Figure 1.**
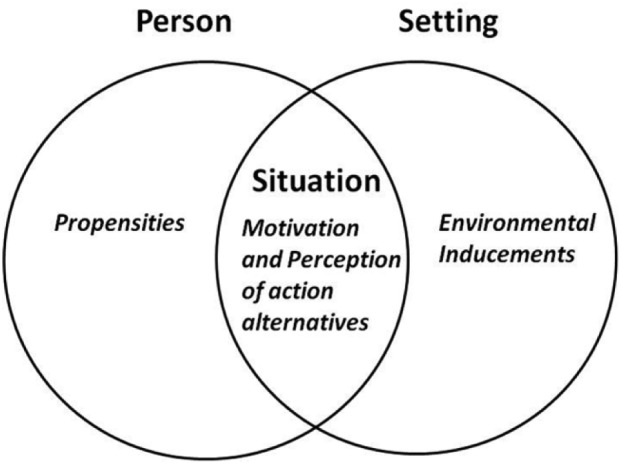
The relationship between person, setting and situation illustrated.

Most leading criminological theory tends to focus on either person-oriented or environment-oriented explanatory factors (although some theories pay lip-service to the importance of both, they typically do not explicate in any detail – or at all – *how* personal and environmental factors interact in the explanation of acts of crime^3^). To bridge this divide, and integrate key insights about the role of people and places in crime causation, we need to focus on situational analysis. While most uses of the term ‘situation’ in criminology seem to refer to the immediate environment and most ‘situational analyses’ seem to differentiate between people *and* situations (see, for example, Birkbeck and LaFree, 1993), SAT maintains that a situation is a particular person’s perception of action alternatives (on which basis he or she makes choices) that emerge when he or she takes part in a certain setting (immediate environment) facing a particular potent motivator. A *situation* is thus not the setting (immediate environment) but the particular *perception of action alternatives* in response to a potent motivator that appear out of a specific person–environment interaction (Figure 1).

SAT insists that acts of crime are *always* an outcome of a ‘person propensity – environmental inducements’ interaction and that crime cannot be properly understood by focusing on just one aspect. A person’s particular crime propensities are triggered by specific features of a setting, and a setting’s particular criminogenic inducements are made relevant by a person’s specific propensities (although the relative importance of a person’s crime propensities and a setting’s criminogenic inducements may vary by circumstance). However, to argue that acts of crime are an outcome of the person–environment interaction does not take us far in explaining *why* crime happens. It is only when we can identify *what* makes people crime prone and *what* makes environments criminogenic, and, crucially, specify *how* their interaction may trigger acts of crime, that we have reached some understanding of the causes of crime.

Against the background that people are rule-guided creatures and that crime is essentially rule-breaking behaviour, SAT proposes that people’s *crime propensity* (the tendency to see and choose acts of crime as an action alternative) is largely dependent on their law-relevant personal morals (internalized rules of conduct, including supporting moral emotions such as shame and guilt) and their ability to exercise self-control (the ability to withstand external pressure to act against one’s own personal morals).^4^ The closer a person’s personal morals correspond to specific rules of conduct stated in the law, the less prone he or she is to violate these rules. The stronger a person’s ability to exercise self-control, the less likely he or she is to be enticed to act contrary to his or her own personal morals. SAT predicts, at one extreme, that people with strong law-relevant personal morals and a strong ability to exercise self-control are largely resistant to momentary criminogenic influences of settings, whereas at the other extreme those who have weak law-relevant personal morals and a poor ability to exercise self-control are vulnerable to momentary influences of criminogenic settings.^5^

The theory proposes that the criminogenic features of a *setting* (the immediate environment a person at any given time can experience with his or her senses) is largely dependent on its (perceived) law-relevant moral context (the moral norms and their enforcement relevant to the motivations – temptations and provocations – people may experience in the setting). Settings (places) are *criminogenic* to the extent that their (perceived) moral norms,^6^ and their level of enforcement, encourage (or do not discourage) acts of crime in response to the opportunities they provide and/or the frictions they create.^7^

### The PEA hypothesis

The situational model of SAT is captured in the *PEA hypothesis* (for example, Wikström, 2014): for any particular motivation (temptation or provocation), the resulting action (**A**), for example an act that constitutes a crime, is an outcome of a perception–choice process (**→**) that results from the interaction (**×**) between relevant personal propensities (**P**) and exposure to relevant setting inducements (**E**).

PxE→A

Motivation is a situational concept. SAT defines *motivation* as ‘goal-directed attention’ and asserts that there are two main kinds of motivators: temptations and provocations.^8^ Temptations arise when there is an opportunity to satisfy a desire or to fulfil a commitment. Provocations occur when a friction (an unwanted external interference) causes anger or annoyance towards the perceived source of the friction or a substitute (see, further, Wikström, 2014: 79). The perception–choice process (→) is crucial for understanding a person’s actions. *Perception* (the selective information we get from our senses) is what links a person to his or her environment, and *choice* (the formation of an intention to act in one way or another) is what links a person to his or her actions (see, further, Wikström, 2006: 76–84). The perception–choice process may be more or less automated (deterministic) – ranging from a fully habitual to a more reasoned process – depending on the circumstances (see, further, Wikström, 2006: 97–9; Wikström, 2014: 80–2; Treiber, 2011). The perception–choice process is most likely to be of a habitual nature when people operate in familiar circumstances with congruent rule-guidance (or are under high levels of stress or emotion) and most likely to involve reasoning when people operate in unfamiliar circumstances or circumstances with conflicting potent rule-guidance. SAT asserts that acts of crime are most likely to happen (being seen and chosen as an acceptable action alternative) when a crime-prone person responds (habitually or deliberatively) to a potent motivator (temptation or provocation) in a criminogenic setting.

According to SAT, *changes* in people’s actions (including their acts of crime) are a result of changes in their personal propensities and/or environmental exposure because such changes affect the input to the perception–choice process that guides people’s action choices (Wikström and Treiber, 2018).

## Key issues in testing the core situational hypothesis of SAT

Based on the more general PEA hypothesis, the main proposition to be tested in this paper is that *young people are differentially vulnerable to criminogenic exposure depending on their crime propensity*. More specifically we hypothesize that young people with a low crime propensity are largely immune to criminogenic exposure whereas young people with a high crime propensity are increasingly vulnerable to criminogenic exposure.

### Measuring spatio-temporal interaction effects

A crucial test of our hypothesis is whether acts of crime are predicted by the *spatio-temporal interaction* between people’s crime propensity and criminogenic exposure. Interaction effects are usually estimated in statistical analysis by studying how the effect of one independent variable (for example criminogenic exposure) on the outcome variable (for example crime involvement) depends on the magnitude of another independent variable (for example crime propensity). Typically the data used in such studies (for example questionnaire data) refer to different scales (for example, one scale measuring criminogenic exposure, one scale measuring crime propensity and one scale measuring crime involvement) that are spatio-temporally unconnected. This is not unproblematic because, even if such a study shows a strong statistical interaction effect (for example, that time spent in criminogenic settings is predictive of the crime frequency only for those with a higher crime propensity), it does not conclusively demonstrate that acts of crime are actually most likely to happen when crime-prone people are in criminogenic settings (because there is no spatio-temporal link in the data between being in a criminogenic setting and committing an act of crime).

Arguably, the ultimate (correct) test of our interactional hypothesis requires exploring whether the elements of propensity, exposure and crime *converge* in time and space. In other words, *do crime-prone people tend to commit acts of crime when they are in criminogenic settings?* To properly test this assumption we need data that locate particular people in particular settings at particular times and tell us whether or not they committed acts of crime in those particular settings at those particular times. The unique space–time budget methodology used in PADS+ helps us achieve this (see further the section on Data and measurements below).

### Crime causation – A question of minutes rather than years

**Figure 2.**
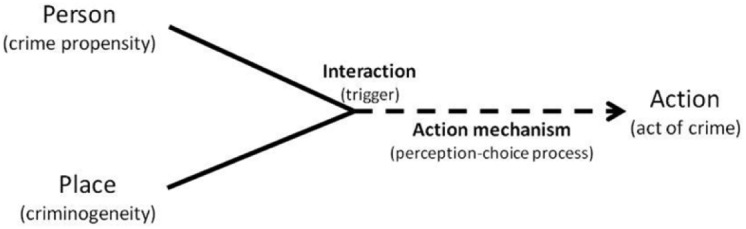
The causal interaction hypothesis illustrated.

SAT asserts that the causes of action, such as acts of crime, are situational. It is the combination of a particular person in a particular setting that triggers (and guides) a perception–choice process that, depending on the input, may or may not encourage an act of crime.^9^ Importantly, there is no causal ordering between propensity and exposure because it is their specific blend that initiates (and guides) the perception–choice process responsible for what action is taken (Figure 2). The *causal time-ordering* is thus between the interaction (‘the trigger’) and the action (‘the outcome’). This is allegedly normally a process of minutes (or in some instances even seconds). The main methodological implication of this is that ideally we should aim to have data (measurements) of people’s propensity, exposure and crimes that are as close as possible in time – preferably measured concurrently.

The importance of time-ordering the ‘cause’ and the ‘effect’ is often stressed as a key element in testing causal hypotheses. However, time-ordering between cause and effect is only *one* necessary but far from sufficient criterion for causation.^10^ The often voiced opinion that longitudinal studies are (always) better than cross-sectional studies when the researcher aims to address questions of causation is highly misguided when examining situational factors because longitudinal data are typically collected on an annual basis. It is hardly an advantage to have annually time-ordered data when exploring *situational* factors and processes; in fact, cross-sectional data are more apt. It would, of course, be advantageous if the trigger–outcome (interaction–action) sequence could be time-ordered, but very few research designs and existing data (including ours) make time-ordering of interaction and action in terms of minutes possible. In practice, therefore, the (second-)best option when studying situational factors and processes is to aim to measure propensity, exposure and crime as close as possible in time (and make the reasonable assumption that the ‘interaction’ produces the ‘action’ rather than the other way around). Space–time budget methodology helps us spatio-temporally link crime propensity, criminogenic exposure and acts of crime.

### Beyond neighbourhoods – Measuring settings and exposure

SAT proposes that only the part of the environment (‘the setting’) that a person experiences with his or her senses can influence his or her perception of action alternatives and choices. Exploring the person–environment interaction therefore requires that we have measures of *settings* (immediate environments) that approximate as closely as possible the part of the environment that a person in a location is likely to directly experience. The aim should be to collect environmental data about the smallest possible geographical units (see, further, Oberwittler and Wikström, 2009). Moreover, since people are not stationary but move around in space, we need a methodology that can also account for their activity fields, that is, their *exposure* to different settings (environments) during the study period. Even if people live in the same house and belong to the same family, they may, depending on their specific way of life, have a widely varying exposure to particular environments – for example criminogenic settings – within and outside their neighbourhood (see, for example, Wikström et al., 2012b: 68).

Most existing studies of the role of the environment and the person–environment interaction in crime causation are based on large (and therefore typically environmentally heterogeneous) geographical areas, and usually the person’s environment is represented only by the area surrounding his or her residence (‘the neighbourhood’). In practice these are therefore studies of how the interaction between people’s characteristics and their neighbourhood characteristics predict their crime involvement (ignoring the wider environment in which a person operates), and usually the data on environments and crime commission are not spatio-temporally linked (for examples, see Simcha-Fagan and Schwartz, 1986; Simons et al., 2014; Wikström and Loeber, 2000). Space–time budget methodology combined with a small area community survey helps us to overcome these problems and advance towards the goal of better measuring the environmental features of settings and people’s activity fields (that is, their specific configuration of exposure to different kinds of settings).

## Data and measurements

The data for this study are taken from the Peterborough Adolescent and Young Adult Development Study (PADS+). This is an ongoing longitudinal study of a cohort of 716 randomly selected boys and girls from the UK city of Peterborough who turned 12 in 2003. The study has so far completed nine waves of data collection. The first wave consisted of one-to-one interviews with each participant’s main care-giver (parent) and the subsequent eight waves consisted of data collection conducted with the participants themselves via one-to-one psychometric tests and space–time budgets, and interviewer-led small group questionnaires. In addition, a range of official records (for example police records and census data) have been collected for the participants and for geographical areas in Peterborough, and two special small area community surveys have been carried out (in 2005 and 2012) with random samples of the Peterborough population aged 18 and older for each *output area* in the city (output areas are small geographical areas with, on average, 300 residents).^11^ For detailed information about the study design, its data and measurements, see Wikström et al. (2012b: 44–106). The data analysed in this paper refer only to Phase 1 of the PADS+ data collection. Phase 1 focuses on adolescence and consists of annually collected data covering the ages 13–17 (the years in which the participants turned 13, 14, 15, 16 and 17). The retention rate of the study is very high and at age 17 (wave 6) 97 percent of the initial sample still took part.

### Space–time budgets

The *space–time budget* methodology is the basis for the spatio-temporal matching of data about people (crime propensities), settings (criminogenic features) and actions (crime).^12^ The space–time budget *places a person in a setting* (output area) at a specific *hour* and thereby directly links the person to the setting spatio-temporally. The space–time budget also collects data about the *circumstances* in which he or she takes part in the setting (for example, with whom and doing what) and data about whether or not he or she committed an *act of crime* when in the setting (thereby directly linking acts of crime and settings spatio-temporally). For details of the space–time budget and its coding, see Wikström et al. (2012b: 67–78, 423–36 and technical appendix A2).

### Small area community survey

Space–time budgets thus allow us to analyse on an hourly basis where young people were (at what geographical locations), in what circumstances (for example, doing what with whom) and whether or not they committed an act of crime. However, they do not provide data about the wider *moral context* (for example, the output area’s level of collective efficacy) in which young people encounter particular circumstances (for example, socialize unsupervised with peers). The space–time budget data have therefore been complemented with relevant environmental data from a specially designed *small area community survey* carried out by the PADS+ research team by a postal survey in the year 2005 (for details, see Wikström et al., 2012a).

The city of Peterborough was (in 2005) divided into 518 output areas (with an average of 125 households). A random sample was drawn from the electoral register of people aged 18 or older for each output area to ensure sufficient data for each of the 518 output areas. It was judged that having a minimum of 10 respondents in each area would be sufficient to achieve reliable measures of output area *collective* features (for further details about and tests of the validity of this argument, see Oberwittler and Wikström, 2009 – see also Snijders and Bosker, 1999). The average number of respondents per output area was 13. In total, 6615 people participated in the 2005 small area community survey, representing an overall response rate of 53 percent (for more details about the small area community survey, its design, reliability and validity, and the content of the questionnaire, see Wikström et al., 2012b: 87–104). The data from the small area community survey are directly linked spatially to the space–time budget at the output area level. However, in contrast to the space–time budget, there is no temporal dimension of the small area community survey.

*Collective efficacy* is a concept that combines residents’ social cohesion and their informal social control potential and aims to measure residents’ willingness to intervene for the common good, such as to prevent disorder and crime (Sampson et al., 1997). We selected collective efficacy as our prime measure of the moral context of a setting because it is an established and tested measure (for example, Duncan et al., 2003; Sampson and Wikström, 2008) with a focus on the level of residents’ enforcement of key common rules of conduct relevant to young people. We used the original questions suggested by Sampson et al. (1997) with one modification.^13^ The Cronbach’s alpha of the combined scales was a highly satisfactory .87. The measure was scaled so that high values meant poor collective efficacy (that is, low social cohesion and weak informal social control). As recommended by Sampson et al. (1997), the scale we use to classify output area levels of poor collective efficacy is an empirical Bayes estimate adjusted for individual-level socio-demographic composition (for details, see Wikström et al., 2012b: 143–6).

### Land-use data

One limitation of the 2005 small area community survey data is that it is largely restricted to residents’ generalized observations of other residents’ relationships and behaviour. Social life and controls in urban areas are determined not only by residents but also by the quantity and characteristics of temporary populations (visitors) that frequent the area and their activities. One kind of urban area that tends to have particularly high levels of visitors and non-residential activities (for example commerce and entertainment) is city and local centres, and because such features are an important aspect of area social life and control we decided to classify, based on land-use data, whether or not an output area was a city or local centre area. As is the case for the small area community survey, these data are spatially linked to the other data but lack a temporal dimension (that is, data about day and hourly variations). We make the assumption that these data satisfactorily represent the relevant impact of visitors and non-residential activities on the social life and control in the output area (although we are aware, of course, that centre area activities tend to be very different at different times of the day, being focused on commerce during the day and on entertainment at night).

### The measure of criminogenic settings

Space–time budget data, complemented with data from the small area community survey and land-use data, provide information about what kinds of settings (circumstances and their moral context) a person has taken part in during the studied periods and their acts of crime.^14^ The criteria in this study for a setting to qualify as *criminogenic for young people* are that *the output area is an area with poor collective efficacy (defined as belonging to the output areas with the 25 percent highest scores of poor collective efficacy) or a city or local centre area* and *the young person taking part in the setting is engaged in unstructured and unsupervised activities with peers*.

### Questionnaires – Measuring crime propensity

To explore the interaction between personal propensity and setting exposure as causes of acts of crime, data about the participants’ crime propensity need to be added. To measure people’s crime propensity we use an index that combines standardized values (*Z*-scores) from a scale of *generalized personal morals* (for details, see Wikström et al., 2012b: 132–5) and a scale of *generalized ability to exercise self-control* (for details, see Wikström et al., 2012b: 135–7).^15^ Previous reviews of research have demonstrated that personal morals (for example, Stams et al., 2006) and ability to exercise self-control (for example, Pratt and Cullen, 2000) are indeed strong predictors of crime involvement.

As previously argued, data about people’s crime propensity should be collected as close as possible in time to the data about setting exposure and crime commission. The data about the participants’ personal morals and abilities to exercise self-control are taken from scales measured on the same occasion as the space–time budget data.^16^ When exploring the person–setting interactions, we matched the 2003 data on participants’ crime propensity with the 2003 space–time budget data, the 2004 data on participants’ crime propensity with the 2004 space–time budget data, and so forth, to make sure that the measurement of people’s crime propensity was temporally linked as closely as possible to the measurement of their criminogenic exposure and crimes. This also means that any age-related (annual) changes over the study period (ages 13 to 17) in a participant’s crime propensity were taken into account when analysing the person–exposure interaction.

## Analytic strategy

To explore how young people’s crime propensity and the proposed criminogenic factors of the environment, in combination, can predict acts of crime, we will use an artificial neural network modelling approach. We will first test the predictive power of crime propensity and the elements making up criminogenic exposure and, then, test the PEA hypothesis by studying how the influence of criminogenic exposure (exposure vs. non-exposure) during a particular hour affects the probability of crime in that same hour for young people with different levels of crime propensity. Finally, and based on the outcome of the analysis of the propensity–exposure interaction, we will estimate the impact of (hypothetical) successful reductions in crime propensity and criminogenic exposure for young people’s crime involvement.

### Artificial neural network modelling

Artificial neural networks (ANNs) are highly flexible tools for performing non-linear regression and classification (for example, Bishop, 2009) that so far have had a rather limited application in criminological studies (for example, Olligschlaeger, 1997). An ANN specifies a mapping between input and output variables of interest; here the inputs are the various factors hypothesized to contribute to the commission of acts of crime, and the output is the probability that a crime is committed in a particular hour. We will select the optimal model – the set of factors that have the greatest predictive power – via cross-validation. That is, we will randomly split the original data set into two parts, the *training data*, which are used to fit the model and estimate its parameters, and the *test data*, which we aim to predict using the fitted model. Each ANN uses a specific set of included factors as the predictors of crime probability per hour. The ANN was cross-validated by training on 50 percent of the available data, with 50 percent withheld for cross-validation.

A model predicts the probability that a crime will be committed, based on the available information provided by the test input. To evaluate the quality of this prediction against the actual crimes in the test output data, we will use the *receiver operating characteristic* (ROC), a canonical test for the predictive power of a binary classifier (Brown and Davis, 2006; Green and Swets, 1966). Setting a threshold probability, and declaring that once this probability is reached we will predict that a crime will be committed, we can test how many true positives (correct identifications of crimes) versus false positives (predictions of crimes when none occurred) the model produces. Varying this threshold alters the balance between true positives and false positives, producing a characteristic curve, the ROC, which shows how the rate of true positives varies with the rate of false positives. The procedure was repeated 10 times with different subsets of the data withheld or used for training to determine the average performance of the ANN.

The area under the ROC curve (AUC) is a single number that quantifies the performance of the classifier. An AUC of 1 indicates that all true positives are identified without any false positives, that is the classifier is perfect. Conversely, an AUC of 0.5 indicates the performance characteristic of a random classifier, that is the classifier is useless. The higher the AUC produced by a model the greater its predictive power.

ANNs that show high predictive power (AUCs) are associated with important factors in predicting the outcome (in our case, the occurrence of crime). The cross-validation method, focusing on the ability to predict data previously unseen by the ANN, identifies models that generalize well and thus avoids overfitting problems common to regression methods. Thus we can attribute increased predictive performance to identifying factors that genuinely affect the probability that an individual will commit a crime.

Factors are selected in a ‘greedy’ manner through an iterative process that tries each element on its own, then fixes the most important, then tries all the others next, and fixes the next most important, and so on. That is, all possible one-factor ANNs are tested, and the best performing is selected. This factor is then included in all future ANNs. Subsequently all combinations of the best single factor and one other factor are tested to find the best two factors, which are subsequently included in all future ANNs, and so forth, until all factors have been included.

The collected data have a time-series structure, being the recorded movements and activities of individuals for each hour over four separate days each year (190,508 total waking hours for analysis, including 125 crime hours). As such, temporal correlations in the data can be expected to be significant and potentially introduce artefacts such as pseudo-replication into our analysis. To account for the time-series nature of the data we always include an autoregressive (AR) component in our models, which controls for the possibility that observed crimes are continuations of those that were initiated in the previous hour (this was actually the case for 10 crime hours). The predictive models are generated as combinations of the other included factors (Table 1). In addition to AR and the proposed causally effective factors (crime propensity and the elements of criminogenic exposure), we also included day of week (D) and time of day (T) as ‘controls’. The rationale for this is that the variables measuring the moral context (collective efficacy, city and local centres) lack a temporal dimension (if our variables are sufficient to measure exposure then we would expect day and hour to have no residual predictive power). With the AR factor fixed as an element of the model, and with eight remaining ‘free’ factors, we can generate 2^8^, that is 256, possible models, though our ‘greedy’ selection algorithm reduces the number of models tested to a more manageable 36.^17^ In each case the output remains the same – the occurrences of crime – but the inputs are chosen by which factors are included in each model.

**Table 1.** Factors included in the ROC analysis and their abbreviations.

*Crime propensity*
PS = Crime propensity score (index of personal morals and ability to exercise self-control)
*Elements of criminogenic exposure*
US = Unsupervised (no adult guardians present)
PPP = Peers present
LC = In local centre
CC = In city centre
USt = Engaged in unstructured activities
CE = In area with poor collective efficacy
*Controls*
AR = Autoregressive component
D = Day of week
T = Time of day

## Findings

### The predictive power of crime propensity and elements of criminogenic exposure

To test the predictive power of crime propensity and the elements making up criminogenic exposure we assessed the value of each factor by using the area under the ROC (AUC) to evaluate the predictive accuracy of a series of ANNs. The value of the AUCs may be interpreted in the following way: *If we take a random pair of hours, one of which contains crime and one of which doesn’t, AUC is the probability that we assign a higher probability of a crime to the hour ‘with’ crime than the one ‘without’ crime based on the included predictors*.

**Figure 3.**
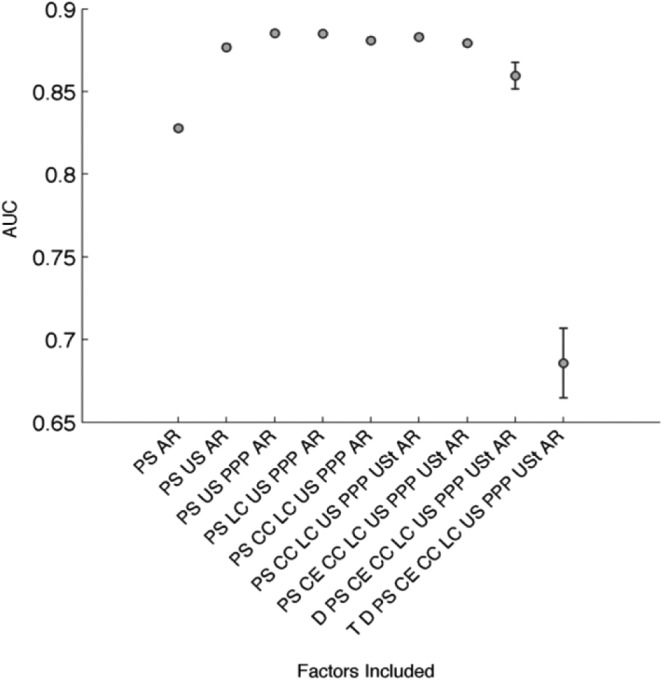
Crime propensity, elements of criminogenic exposure and controls. Analysis of predictive performance by area under ROC curve (AUC). *Note*: See Table 1 for the key to the abbreviations.

Our findings show a number of clear facets (Figure 3). Crime propensity (PS) is selected first, and has the bulk of the predictive power. Subsequently all the elements that constitute our proposed exposure variable are selected, with an initial substantial increase in predictive power for being unsupervised (US) and a subsequent plateau without any significant drop in prediction for any of the additional elements (PPP, LC, CC, USt, CE) that constitute our measure of criminogenic exposure.

The findings support the use of our proposed criminogenic exposure variable (that is, taking part in unsupervised and unstructured activities with peers in a place of poor collective efficacy or in the city or a local centre) as a measure of the criminogenic exposure of a setting. Day of the week (D) and time of day (T), which were included as controls, lead to initially small (D), then large (T) significant *decreases* in predictive power (confidence intervals for day and time shown in Figure 3). This is evidence of overfitting, and suggests that, once crime propensity and criminogenic features of the setting (moral context and circumstances) are accounted for, day and time have no predictive value and that the included elements of criminogenic exposure pick up the day and time variations in crime (consequent on the view that they are markers of criminogenic features rather than factors with any causal efficacy). All in all, our measures of crime propensity and criminogenic exposure are strong predictors of the probability of committing acts of crime in a given hour.

### The interaction between crime propensity and criminogenic exposure in predicting the probability of crime in a particular hour

**Figure 4.**
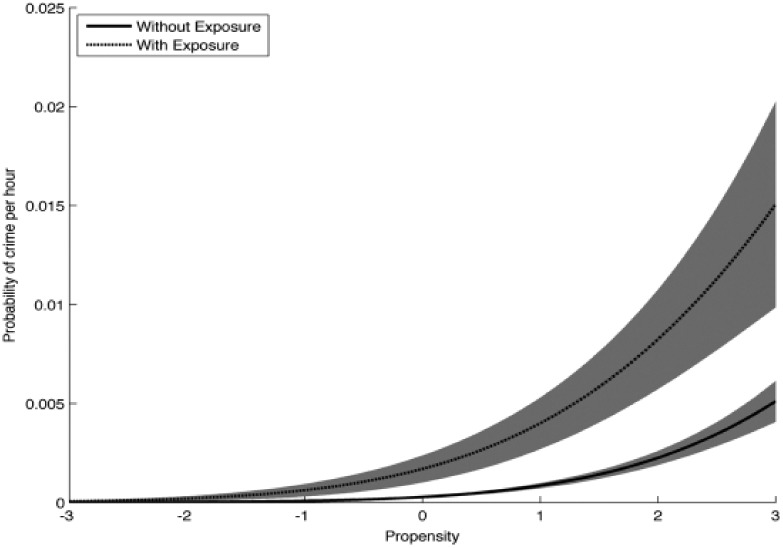
Probability of crime per waking hour with and without criminogenic exposure by crime propensity.

Having established the efficacy of the elements of the criminogenic exposure variable, alongside the predictive power of crime propensity, we now train an ANN on two factors, using 100 percent of the data: crime propensity and the composite criminogenic exposure variable (that is, unsupervised with peers taking part in unstructured activities in a residential area with poor collective efficacy or in the city or a local centre). We use this to calculate directly *the probability that an individual with a given crime propensity will commit an act of crime when exposed or not*. To do this we feed the trained ANN with a range of values of (standardized) crime propensity from −3 to 3 and of criminogenic exposure with a value of 1 or 0 (exposed vs non-exposed). The predicted crime probabilities (that is, the probability that a crime will occur in a particular hour) by level of crime propensity are plotted for hours criminogenically exposed versus unexposed in Figure 4.

This result shows several crucial features. Firstly, the probability of committing a crime is strongly, and non-linearly, related to crime propensity (the shaded areas in Figure 4 indicate the 95% confidence intervals). Young people with a high crime propensity commit a disproportionate number of crimes. Even within the group with a high crime propensity (PS > 1), those with the *highest* propensity are committing a much larger number of crimes per hour than their high propensity peers. Secondly, exposure to criminogenic settings results in a substantial increase in crime probability. This increase is, in absolute terms, much higher for those with a high crime propensity. Therefore crimes are disproportionately committed by high crime propensity people in criminogenic settings. Thirdly, while the estimated probability of committing a crime for young people with a low crime propensity is also higher when in a criminogenic setting, the difference in absolute terms for these young people is negligible (the difference is not statistically significant when crime propensity is less than −1.5, as indicated by the fact that the confidence intervals overlap). Our findings thus give strong support for the PEA hypothesis: acts of crime are most likely when crime-prone young people take part in criminogenic settings.^18^ Moreover, our findings specify this relationship: the higher a young person’s crime propensity the stronger the influence of criminogenic exposure on his or her probability of committing an act of crime. The interaction of people and places clearly matters in crime causation.

### A note on the potential problem of ‘floor effects’

Our interactional analysis is based on modelling 125 crime events across about 190,000 hours. This fact may cause some colleagues to worry whether our findings are largely a result of the distribution of the crime variable and reflect only a (so-called) floor effect (for example, Osgood, 2002: 337–40). However, there is no floor effect inherent in our classification methodology. Our neural network predicts the probability that a crime will occur or not within a single hour – a binary variable. This is fitted by maximizing the likelihood of the observed crime/no crime events in the observed hours based on the parameters of the neural network (via back-propagation, a gradient descent algorithm for neural networks – see Rumelhart et al., 1986), not via any least-squares method or similar.

Our evidence for an interaction effect is simply that the probability of crime rises with crime propensity, as shown in Figure 4, but that it is always higher when there is (stronger) criminogenic exposure. Since the two curves in Figure 4 are not separated by a constant, but instead exhibit a roughly constant ratio, this is most parsimoniously explained by criminogenic exposure and crime propensity interacting – rather than just summing, as per the standard linear model.

### Changes in crime as a result of changes in crime propensity and criminogenic exposure

According to SAT, to change people’s crime one can either change people’s crime propensities or their criminogenic exposure^19^ (or both) because such changes alter the person–environment interaction, which is the input to the perception–choice process that affects whether or not a person is likely to see and choose an act of crime as an action alternative in response to potent motivators (Figure 2). For illustrative purposes we have calculated how much reduction in crimes per 1000 hours would result if one succeeded in reducing all young people’s crime propensity, or all young people’s criminogenic exposure, by 1 standard deviation (STD) (Table 2). These calculations are based on the relationships between crime propensity, criminogenic exposure and probabilities of crime, as shown in Figure 4. The result suggests that successfully reducing young people’s crime propensity (as defined here) has a much greater effect on their crimes than reducing their criminogenic exposure (as defined here), although both changes would lead to reductions in crime.

**Table 2.** Crimes per 1000 waking hours (change following propensity or exposure reduction).

	Crimes per 1000 hours	Change crimes per 1000 hours	Percent crime reduction
Base rate	0.62		
−1 STD propensity	0.23	−0.39	−62.9
−1 STD exposure	0.53	−0.09	−14.5

## Conclusions and discussion

In this paper we have advocated SAT as a theory that effectively integrates key insights from people- and place-oriented criminological theory and research, and we have tested its core situational hypothesis (the PEA hypothesis) using a unique set of data from the Peterborough Adolescent and Young Adult Development Study (PADS+). The PADS+ data and methodologies are exceptionally suitable for conducting situational analysis because they enable detailed spatio-temporal matching of people, places and acts of crime, and provide in-depth data about the circumstances and environments in which young people with different crime propensities take part (and commit acts of crime). In the present study we have analysed more than 190,000 hours (time awake) of people (propensities) and place (environmental inducements) convergences in adolescence (ages 13 to 17).

SAT proposes that humans are essentially rule-guided actors and that the defining feature of acts of crime is that they breach rules of conduct (stated in law) and, therefore, law-relevant personal morals and the ability to exercise self-control are the key individual characteristics upon which a person’s crime propensity is dependent. The findings from our study strongly support this assertion by demonstrating that crime propensity (measured as an index of law-relevant personal morals and the ability to exercise self-control) strongly predicts the probability of committing an act of crime.

However, SAT also stresses that acts of crime are *always* an outcome of the interaction between a person’s crime propensity and his or her criminogenic exposure, where criminogenic exposure is seen as dependent on the moral context of the setting and the circumstances in which people take part in that context. Our findings show that our measures of criminogenic exposure are strong predictors of the probability of crime (which add substantially to the predictive power of crime propensity) and, importantly, that the probability of crime is strongest when a crime-prone person (as defined here) takes part in a criminogenic setting (as defined here). Crucially, we find that *the higher a young person’s crime propensity the more vulnerable he or she is to influences from criminogenic exposure*.

Although our study provides a methodologically and statistically rigorous test of and supports the interactional hypothesis of SAT, it does not examine the proposed action mechanism: the presumed perception–choice process that links the person–environment interaction (the trigger) to the action (the outcome). However, previous research from PADS+ using randomized scenarios regarding the intended use of violence (varying by levels of provocation and supervision) lends some support to this assumption, showing that those with a high crime propensity (measured in the same way as in this study) were more likely to choose a violent response (especially at lower levels of provocation) than those with a low crime propensity (Wikström et al., 2012b: 364–402).

The fact that crime propensity is a stronger predictor of the probability of crime than criminogenic exposure (Figure 3), and that successful reductions in crime propensity seemingly lead to larger reductions in crime than successful reductions in criminogenic exposure (Table 2), is not unexpected since (as our findings show) criminogenic exposure largely affects the probability of crime for those with some level of crime propensity, and particularly (and increasingly) for those with a high crime propensity, whereas it has little or no impact on those with a low crime propensity.

Our results suggest which key personal and environmental factors are implicated in crime causation and, therefore, which factors should be the prime target for crime prevention policy and intervention. However, our findings say nothing about which are the most effective *policies and interventions* to affect people’s crime propensities and criminogenic exposures. They only suggest that *if* we can devise successful policies and intervention to affect people’s crime propensities (by influencing their law-relevant personal morals and abilities to exercise self-control), and successful policies and interventions to affect people’s criminogenic exposure (by influencing the law-relevant moral norms of a setting and their enforcement and/or people’s access to criminogenic settings), policies and intervention that successfully reduce people’s crime propensity would most likely be the more effective.^20^

In this paper the focus has been on situational analysis. Situational analysis explains why crime happens; it specifies the interactions and action mechanisms involved and tells us what moves people to action (what causes action) and what factors are important in that process. However it does not tell us much about how particular criminogenic situations (interactions) come about and what (cultural and structural) factors and processes are important in this respect. SAT suggests that this is fundamentally a question of how *processes of selection* (contemporaneous processes of rules- and resource-based social selection, and agency-based self-selection within the constraints of social selection) create criminogenic person–environment interactions (Wikström, 2014: 84).

Moreover, situational analysis does not tell us why people develop different crime propensities (dependent on their law-relevant personal morals and abilities to exercise self-control), and why places come to vary in their criminogeneity (dependent on their law-relevant moral norms and their enforcement). SAT suggests that this is largely a question of *emergence* (that is, the process by which something becomes as it is). SAT asserts that people’s crime propensities are largely an outcome of *psycho-social processes* of person emergence, particularly processes of moral education and cognitive nurturing of relevance to people’s law-relevant personal morals and abilities to exercise self-control (Wikström and Treiber, 2018). SAT further suggests that the criminogeneity of a place is largely an outcome of *socio-ecological processes* of social emergence, particularly processes of population and activity spatial and temporal differentiation that are of relevance to (time and) place-based law-relevant moral norms and their enforcement (see, further, Wikström, 2011).

Situational analysis is arguably the core of the study of crime and its causes. However, to give a comprehensive account of the question of crime causation, situational analysis needs to be complemented with analyses and investigations of how (as the ‘causes of the causes’^21^) contemporaneous processes of social and self-selection and past processes of social and person emergence are implicated in the creation of the situations that cause acts of crime.

## Notes

1. Criminological theories rarely specify a theory of action (a theory about what moves people to action). When they do, they typically allude to some version of rational choice theory (RCT). However, we do not believe that RCT is a fruitful or satisfactory basis for integrating people- and place-oriented insights about crime causation (for a discussion, see Wikström and Treiber, 2016).

2. See, for example, Hirschi (1969), social bonds; Gottfredson and Hirschi (1990), self-control; Felson (2008), routine activity; Cornish and Clarke (2008), rational choice.

3. Further on this argument, see, for example, Wikström and Treiber (2007), re. self-control theory; and Wikström and Treiber (2016), re. opportunity theory, that is, routine activity and RCT.

4. Please note that SAT’s conceptualization of self-control differs from that in Gottfredson and Hirschi’s (1990) General Theory of Crime (for a discussion, see Wikström and Treiber, 2007). SAT carefully distinguishes between (i) the ability to exercise self-control and (ii) exercising self-control. The former is an individual characteristic; the latter a person’s active management of a situation.

5. It should be stressed that SAT acknowledges that people’s crime propensity may vary significantly depending on the type of crime in question. To adequately study the causes of specific crimes (rule-breakings) one needs to study the specific propensities and exposures that may trigger and guide actions that break that particular rule of conduct.

6. The concepts of ‘place’ and ‘setting’ are closely related and overlapping. According to SAT, the difference is that ‘place’ refers to a specific location in time and space and its immediate environment (objects, people, events), whereas ‘setting’ refers to the part of the immediate environment a person in a specific location experiences with his or her senses.

7. If a setting has a (perceived) high level of enforcement of moral norms promoting acts of crime in response to its particular opportunities or frictions, this enforcement is criminogenic.

8. Motivation is the reason for action. However, people commit particular acts of crime for all kinds of reasons.

9. SAT applies a ‘soft’ version of causality by acknowledging that there are elements of determination (habit) and ‘free will’ (deliberation) in the process when people make choices, although SAT insists that ‘free will’ is always exercised within the constraints of the action alternatives perceived (see, further, Wikström, 2006, 2014).

10. Causation implies a regularly occurring (asymmetric) association between time-ordered variables that holds universally (or at least in a specific context or in certain circumstances) and that makes it possible to predict one (the effect) from the other (the cause). However, the idea of causation is not only the idea of a regular association (and the possibility of prediction) but, crucially, also the idea that the cause in some way produces the effect through a causal process that links the cause to the effect (see, for example, Bunge, 2004; Psillos, 2002).

11. The neighbourhood units in criminological studies typically have average population sizes in the range of 4000–8000 people (for examples, see Bellair, 1997; Sampson et al., 1997; Sampson and Wikström, 2008; Smith and McVie, 2003; Wikström and Loeber, 2000).

12. The space–time budget methodology was first used in criminology in the Peterborough Youth Study (Wikström and Butterworth, 2006), a cross-sectional forerunner to PADS+, in which the technique was piloted and later refined for inclusion in PADS+.

13. We dropped one item asking about the residents’ likelihood to intervene if a local fire station was threatened with budget cuts from the original informal control scale because it significantly reduced the scale’s internal consistency.

14. Which particular circumstances help make a setting criminogenic may depend on the specific age(s) and/or type(s) of crime under study.

15. Each scale was standardized over the five included years (ages). The self-control scale is a modified version of the Grasmick et al. (1993) scale, and the scale of law-relevant morality is largely based on a scale from the Pittsburgh Youth Study.

16. The space–time budget data refer to the days immediately preceding the time of the data collection. There is no reason to believe that people’s crime propensities (based on their personal morals and ability to exercise self-control) will display any significant change over such a short period of time (other than in very extreme cases). The *annual* correlations of the index of crime propensity for the five included years varies between 0.69 and 0.76 (Wikström et al., 2012b: 138).

17. The algorithm reduces the number of models tested to 36 by selecting each factor iteratively. Hence, for example, we investigate only two factor models that include auto-regression, the factor selected for a single factor model. Likewise, our search over three factor models covers only those models incorporating both auto-regression and individual propensity, and so on. This means that we search over 8 + 7 + … 1 = 36 models in total, as the number of remaining factors to be selected from at each stage decreases by one.

18. There is also an increase in absolute terms in the probability of crime by level of crime propensity for settings classified as ‘other’ but not as dramatic as for those defined as criminogenic (please recall that our classification of a criminogenic setting is rather conservative and that the settings classified as ‘other’ may included several of the criminogenic elements jointly defining a criminogenic setting).

19. Reductions in criminogenic exposure can be accomplished either by interventions reducing the criminogeneity of the settings a person takes part in and/or by interventions that limit his or her access to such settings (places).The latter is accomplished mainly by influencing processes of social and self-selection (or through incapacitation).

20. Developmentally, exposure and propensity are not unrelated, with, for example, the implication that, in the longer term, policies and interventions targeting exposure may also affect people’s crime propensities.

21. Causes of the causes are causally effective factors and processes further back in a causal chain, for example factors and processes that form and shape a person’s crime propensities (see Wikström, 2011).
